# Vagus nerve stimulation (VNS)-induced layer-specific modulation of evoked responses in the sensory cortex of rats

**DOI:** 10.1038/s41598-020-65745-z

**Published:** 2020-06-02

**Authors:** Hirokazu Takahashi, Tomoyo I. Shiramatsu, Rie Hitsuyu, Kenji Ibayashi, Kensuke Kawai

**Affiliations:** 1 Department of Mechano-informatics, Graduate School of Information Science and Technology, The University of Tokyo, Tokyo, Japan; 2 Department of Neurosurgery, Graduate School of Medicine, The University of Tokyo, Tokyo, Japan; 3 Department of Neurosurgery, Jichi Medical University, Tochigi, Japan

**Keywords:** Cortex, Epilepsy

## Abstract

Neuromodulation achieved by vagus nerve stimulation (VNS) induces various neuropsychiatric effects whose underlying mechanisms of action remain poorly understood. Innervation of neuromodulators and a microcircuit structure in the cerebral cortex informed the hypothesis that VNS exerts layer-specific modulation in the sensory cortex and alters the balance between feedforward and feedback pathways. To test this hypothesis, we characterized laminar profiles of auditory-evoked potentials (AEPs) in the primary auditory cortex (A1) of anesthetized rats with an array of microelectrodes and investigated the effects of VNS on AEPs and stimulus specific adaptation (SSA). VNS predominantly increased the amplitudes of AEPs in superficial layers, but this effect diminished with depth. In addition, VNS exerted a stronger modulation of the neural responses to repeated stimuli than to deviant stimuli, resulting in decreased SSA across all layers of the A1. These results may provide new insights that the VNS-induced neuropsychiatric effects may be attributable to a sensory gain mechanism: VNS strengthens the ascending input in the sensory cortex and creates an imbalance in the strength of activities between superficial and deep cortical layers, where the feedfoward and feedback pathways predominantly originate, respectively.

## Introduction

Vagus nerve stimulation (VNS) that applies electric pulses to the vagus nerve at regular intervals has demonstrated therapeutic efficacy in alleviating refractory epilepsy^1,2^ and depression^3,4^. Multiple studies have characterized the various neuropsychiatric effects of VNS^5,6^: decreased nociceptive thresholds^7^, enhanced memory and cognition^8–11^, attenuated anxiety^12^, augmented fear extinction learning^13^, guided advantageous decision-making^14^, alleviated consciousness disorder in vegetate-state patients^15^, and impaired cognitive flexibility and creativity^16^. VNS also gates learning-induced plasticity in the sensory^17^ and motor cortices^18,19^; this accounts for the use of VNS as a therapeutic option to treat neural disorders, such as tinnitus^20,21^. As VNS anatomically activates both the locus coeruleus and dorsal raphe, which release noradrenaline (NA) and serotonin (5-HT), respectively^22,23^, and exerts modulation in the basal forebrain area, which releases acetylcholine (ACh)^24,25^, the neuropsychiatric effects are likely mediated by these neuromodulators. While the global modulation of multiple afferent fibers in the central nervous system has been implicated in the mechanisms underpinning VNS-induced neuropsychiatric changes^6,26–28^, the detailed effects of VNS at the level of cortical microcircuit remain unknown.

The relatively denser innervation of superficial layers by NA, 5-HT and ACh nerve terminals^29–36^ and the comparative abundance of DA terminals in deep cortical layers^37–39^ informs our hypothesis that the neuromodulation of VNS is layer-specific. Such layer-specific neuromodulation might contribute to the balanced integration of bottom-up and top-down sensory inputs^40–42^, which might underlie various neuropsychiatric effects. Supporting this hypothesis canonical motifs of inter-layer microcircuits indicate that the feedforward pathway, i.e., from the primary sensory area to the higher-order area, predominantly originates from the supragranular layers, while the feedback pathway from infragranular layers^43–47^.

To test our hypothesis, we characterized laminar profiles of auditory-evoked potentials (AEPs) in the primary auditory cortex (A1) of anesthetized rats with an array of microelectrodes. We predicted that VNS enhances AEP selectively in the superficial layers of the A1. We also investigated whether VNS affected stimulus-specific adaptation (SSA), which plays substantial roles in maximizing the saliency and sensory resources of unexpected stimuli in complex and changing environments^48–53^. We predicted that relative weakening of feedback pathway diminishes the history-dependent prediction indexed by SSA. Thus, we propose that VNS involves a sensory gain control mechanism that enhances ascending cortical inputs.

## Results

**Figure 1:**
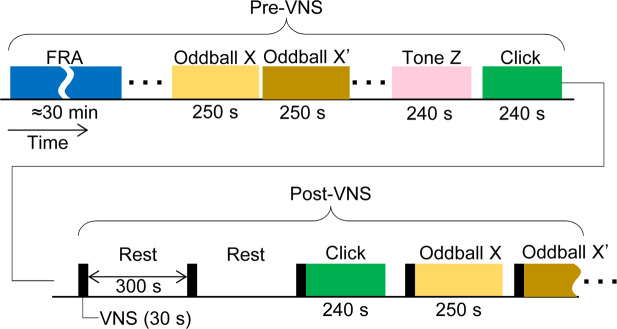
Experimental procedure. The experiments consisted of two main conditions: pre- and post-VNS. The pre-VNS sessions completed prior to VNS and always preceded the post-VNS sessions to avoid confounding the effect of VNS. During post-VNS sessions, 30-s period of VNS was made with an interval of 300 s (5 min). AEPs were characterized in the click sequence, tone sequence, oddball paradigm, etc., each test block of which was designed to be shorter than 5 min. Each oddball paradigm consisted of 2 blocks; in the second block (oddball X′), the tones frequencies of standard and deviant stimuli were inverted from those in the first block (oddball X). Tone sequences of an arbitrary tone burst (Tone Z), whose frequency was close to CF at the test shank, were used to characterize CSD. Prior to the main experiments, we characterized FRA and identified the CF at each of the recording sites in the auditory cortex and thalamus. VNS, vagus nerve stimulation; AEP, auditory evoked potential; FRA, frequency response area; CF, characteristic frequency; CSD, current source density.

**Figure 2:**
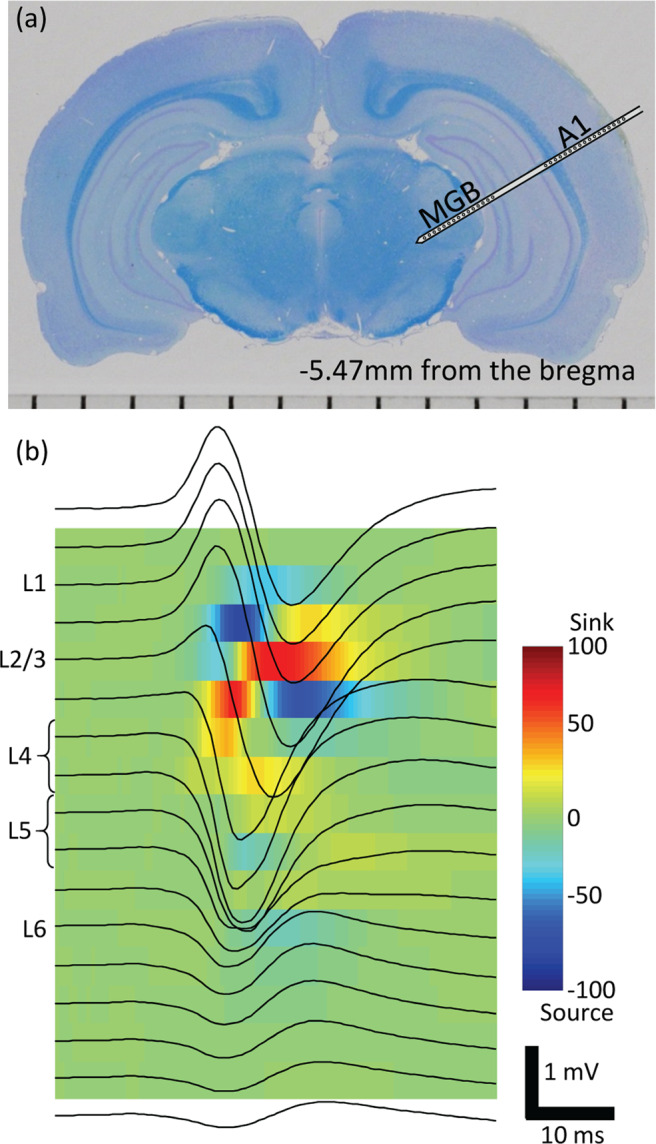
Electrophysiological experiments. (**a**) Recording with microelectrode array in the A1 and MGB. A coronal histological section of a representative test animal. (**b**) Laminar recording and current source density analysis in the A1. Upon recording the AEPs across A1 layers (black traces on the image), the CSD analysis was performed to locate the test sites in L1, L2/3, L4, L5 and L6. A1, primary auditory cortex; MGB, thalamus; AEP, auditory evoked potential; CSD, current source density; L, layer.

**Figure 3:**
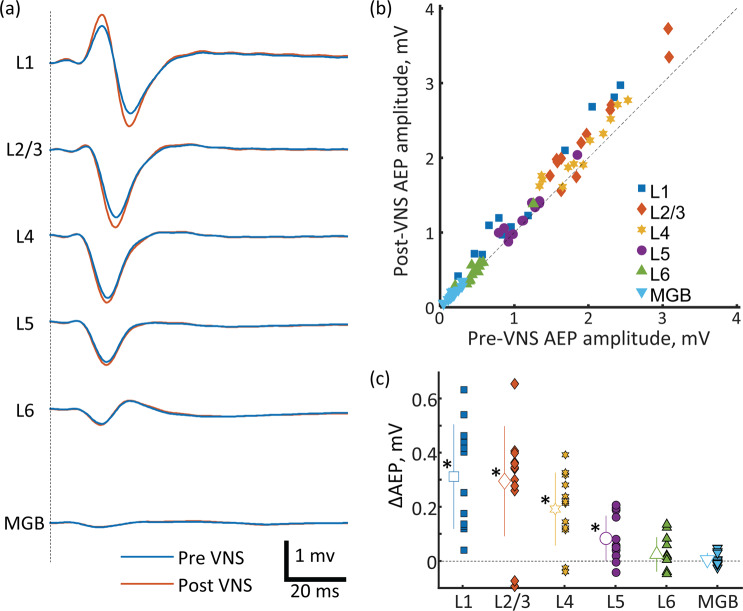
Click sequences. (**a**) AEPs across A1 and in MGB layers for the pre- and post-VNS conditions. The present AEPs were grand-averages across test animals. (**b**) Pre- vs. post-VNS AEP amplitudes. (**c**) Layer-specific increase of AEP amplitude following VNS. Outline markers with error bars indicate the means with standard deviations. AEP, auditory evoked potential; A1, primary auditory cortex; MGB, thalamus; VNS, vagus nerve stimulation. * indicates statistical significance (p < 0.05).

**Figure 4:**
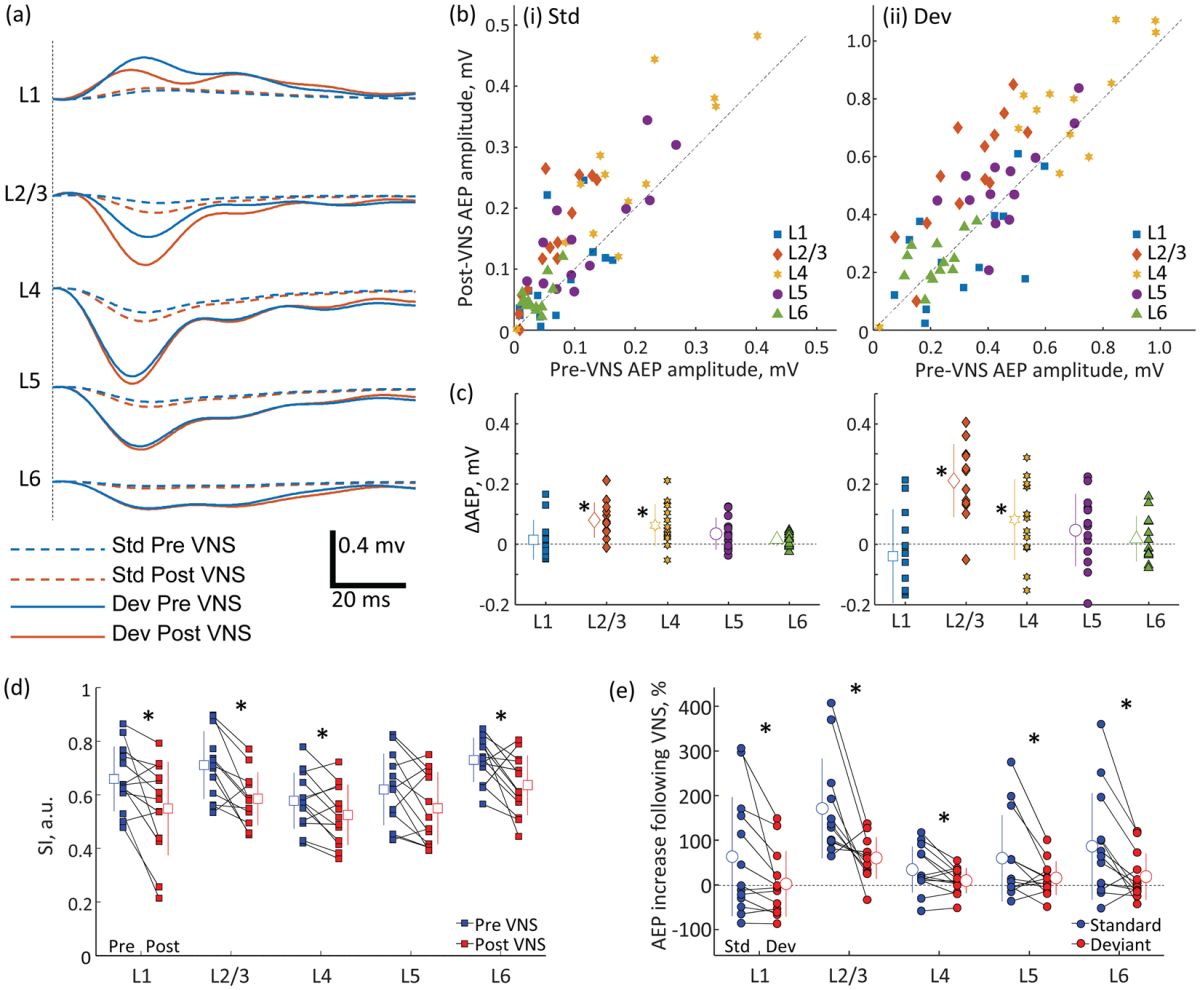
Oddball paradigm. (**a**) AEPs across layers in A1. Standard (Std)- and deviant (Dev)-evoked AEPs in either the pre- or post-VNS condition are presented. The present AEPs were grand averages across test animals. (**b**) Pre- vs. Post-VNS AEP amplitudes: (i) Std; (ii) Dev. (**c**) Layer-specific increase of AEP amplitude following VNS. Outline markers with error bars indicate the means with standard deviations. (**d**) SI across layers under pre- and post-VNS conditions. (**e**) VNS effects on Std- and Dev-evoked AEPs across layers. The proportional increases of AEPs following VNS were quantified. VNS-induced increases of standard-evoked AEPs were larger than those of deviant evoked AEPs, resulting in the decrease of SSA. AEP, auditory evoked potential; A1, primary auditory cortex; VNS, vagus nerve stimulation; SI, stimulus specific adaptation index; SSA, stimulus specific adaptation. * indicates statistical significance (p < 0.05).

One week post-implantation of VNS system, we compared AEPs without VNS (pre-VNS condition) to those following VNS (post-VNS condition) in isoflurane-anesthetized rats (Fig. 1). The main experiments consisted of a click sequence and oddball paradigms of tone bursts, where test stimuli were presented at a rate of 1 Hz and AEPs were grand-averaged across trials in each session^53^. Prior to the main experiments, the characteristic frequency (CF) were routinely characterized at each recording site to determine test tone frequencies near CF in the oddball paradigm. A custom-made microelectrode array electrophysiologically characterized AEPs in every cortical layer of A1 simultaneously with local field potentials (LFPs) in the thalamus – i.e., the ventral division of medial geniculate body (MGB)^54^. The microelectrode array had three shanks, each of which had 15 distal sites in the MGB and 17 proximal sites in the A1 (Fig. 2a). The present data were obtained from 12 shanks in the A1 and 14 recording sites in the MGBs of seven animals. Based on the current source density (CSD) analysis, the recording sites were classified into separate layers of the A1: layers 1, 2/3, 4, 5 or 6 (L1, L2/3, L4, L5, or L6) (Fig. 2b). AEPs exhibited the largest negative peak in L4 (Figs. 3a and 4a) and similar negative peaks in L2-L6. On the other hand, AEP morphology in L1 usually had a positive peak as the first component. We therefore characterized the AEP amplitude as the first positive peak in L1 and the first negative peaks in L2–L6 for the following analyses.

### Click sequence

We first investigated whether and how VNS modulated AEP in the click sequence (presented at a rate of 1 click/s). The comparison between the pre- and post-VNS conditions revealed that VNS is more likely to increase AEP amplitude in superficial layers than in deep layers (Fig. 3a). A two-way repeated measures ANOVA, with VNS condition and layers as factors, indicated a significant main effect of VNS on AEP amplitude (F = 24.179, df = 1, p < 0.001) and a significant interaction between layer and VNS (F = 3.976, df = 1, p = 0.049) (Fig. 3b). Post hoc comparisons showed that VNS increased the AEP amplitude in L1 (two-tailed paired t-test throughout, t = 5.613, df = 11, p = 0.0002), L2/3 (t = −5.046, df = 11, p = 0.0004), L4 (t = −4.960, df = 11, p = 0.0004), and L5 (t = −3.490, df = 11, p = 0.0051) but did not influence the AEP amplitudes in L6 (t = −1.372, df = 11, p = 0.1974). The VNS-induced increase in AEP amplitude (ΔAEP) was not uniform across cortical layers (Fig. 3c). The effect size of VNS on the AEP amplitudes was different across cortical layers: the magnitude of the AEP modulation was similar between L1 (paired Cohen’s d = 1.62), L2/3, (Cohen’s d = 1.45) and L4 (Cohen’s d = 1.43), but decreased across L5 (Cohen’s d = 1.04) and L6 (Cohen’s d = 0.39).

To confirm that the VNS-induced increase of AEP amplitude was produced in the cortex and not at the level of the thalamus, we investigated whether the VNS modulated AEP in the thalamus (MGB). Consequently, the thalamus showed no significant effect of VNS on AEP amplitude (MGB in Fig. 3a–c) (t = −0.866, df = 13, p = 0.4022, Cohen’s d = 0.23).

### Oddball paradigm

We then characterized AEP in the oddball paradigm of tone bursts (presented at 2 tones/s). Standard and deviant stimuli had different frequencies that are 3/5 octave apart and were presented with a probability of 90 and 10%, respectively. Consistent with the results for the click sequences, VNS was found to be more likely to increase AEP in superficial layers than in deep layers in response to both standard and deviant stimuli.

For the standard stimuli (Fig. 4b[i]), a two-way repeated measures ANOVA with VNS condition and layers as factors revealed a significant main effect of VNS on AEP amplitude (F = 13.104, df = 1, p < 0.001) but no interaction between VNS and layers as factors (F = 1.560, df = 1, p < 0.215). Post hoc analysis showed that VNS increased the AEP amplitude of standard stimuli in L2/3 (two-tailed paired t-test throughout; t = −4.825, df = 11, p = 0.0004) and L4 (t = −2.952, df = 11, p = 0.0121) but did not affect L1 (t = 0.581, df = 11, p = 0.5721), L5 (t = −1.739, df = 11, p = 0.1076), or L6 (t = −1.488, df = 11, p = 0.1627) (Fig. 4c[i]). The magnitude of the AEP modulation was highest for L2/3 (paired Cohen’s d = 1.33) and decreased across L4 (Cohen’s d = 0.81), L5 (Cohen’s d = 0.48), and L6 (Cohen’s d = 0.41). L1 also showed a small size effect (Cohen’s d = 0.16).

For the deviant stimuli (Fig. 4b[ii]), the two-way repeated measures ANOVA showed a significant main effect of VNS on AEP amplitude (F = 11.056, df = 1, p = 0.001). The interaction between layer and VNS was marginally non-significant (F = 3.835, df = 1, p = 0.053). Post hoc analysis revealed that VNS increased the AEP amplitude in L2/3 (t = −4.644, df = 11, p = 0.0006) and L4 (t = −3.053, df = 11, p = 0.01) but only marginally affected L5 (t = −2.032, df = 11, p = 0.0649) (Fig. 4c[ii]). No effects of VNS on L1 (t = −0.024, df = 11, p = 0.9816) or L6 (t = −1.804, df = 11, p = 0.0964) were observed. The magnitude of the peak amplitude modulation was highest for L2/3 (paired Cohen’s d = 1.28) and decreased across L4 (paired Cohen’s d = 0.85), L5 (Cohen’s d = 0.56), and L6 (Cohen’s d = 0.50). L1 also showed a small size effect (Cohen’s d = 0.007).

These effects of VNS on AEP amplitude also affect SSA (Fig. 4d), which was quantified by SSA index (SI): ${SI}=({Pd}-{Ps})/({Pd}+{Ps})$, where Ps and Pd are the peak amplitudes of the standard and deviant AEPs, respectively. A two-way repeated measures ANOVA with layer and VNS as factors showed a significant main effect of VNS on SI (F = 7.218, df = 1, p = 0.008) but no interaction between the factors (F = 2.979, df = 1, p = 0.088). Post hoc paired t-tests revealed that VNS decreased SI in L1 (t = −2.866, df = 11, p = 0.0142, Cohen’s d = −0.795), L2/3 (t = −2.320, df = 12, p = 0.038, Cohen’s d = −0.643), L4 (t = −2.298, df = 12, p = 0.0404, Cohen’s d = −0.637) and L6 (t = −2.600, df = 12, p = 0.0232, Cohen’s d = −0.721), and only marginally affected L5 (t = −2.121, df = 12, p = 0.0555, Cohen’s d = −0.588). The decrease in the SI following VNS was more likely caused by selective increases in the AEP amplitudes to the standard stimuli than those to the deviant stimuli. For standard- and deviant-evoked AEPs, we quantified the proportional increases in the AEP amplitude following VNS – i.e., ΔAEP/Pre-VNS AEP amplitude (Fig. 4e) – and found that standard-evoked AEPs exhibited larger proportional increases in amplitude than deviant-evoked AEPs in L1 (t = 2.478, df = 12, p = 0.029, Cohen’s d = 0.57), L2/3 (t = 3.462, df = 12, p = 0.005, Cohen’s d = 0.91), L4 (t = 2.443, df = 12, p = 0.031, Cohen’s d = 0.59), L5 (t = 2.276, df = 12, p = 0.0420, Cohen’s d = 0.60), and L6 (t = 2.958, df = 12, p = 0.0120, Cohen’s d = 0.74).

Thus, although VNS increased both standard and deviant AEP, the effects on standard AEP were significantly larger than those on deviant AEP, leading to the decrease of SSA.

## Discussion

Investigating the effects of VNS on AEPs within the cortical layers of the A1, we found that VNS increased AEP amplitudes in the superficial layers (L1–L4) and that this effect diminished with cortical depth (L5 and L6). An oddball paradigm demonstrated that VNS had a proportionally larger effect on the increase of AEP amplitudes in response to standard stimuli relative to deviant stimuli. This proportional increase resulted in a decreased SSA across all layers of the auditory cortex.

Layer-specific modulation is reportedly involved in higher-order brain functions; e.g., attention dominantly suppresses and enhances responses in the superficial layer (L2/3) and middle-deep layers (L4–6), respectively^55^. The attention-induced modulation of cortical oscillation also varies across layers^56^, and active locomotion modulates membrane potentials and stimulus-evoked spike activities in a layer-specific manner^57,58^. For example, whisking suppresses somatostatin-positive (SST+) interneurons in L2/3, but activates L4^59^. Learning-induced plasticity is more predominant in the superficial layer than in the deep layer^60–62^. Such layer-specific neural encoding and plasticity are likely enabled by both layer-specific neuromodulation and inter-layer microcircuits. Thus, the layer-specific modulation of the sensory cortex by VNS could account for its neuropsychiatric effects.

We anesthetized animals during experiments with isoflurane, which exerts profound effects on neural activities^63^. Specifically, the anesthesia exerts antagonistic effects on the excitatory NMDA receptor, agonistic effects on the inhibitory GABA_A_ receptor, and attenuates the feedback pathway. The influences of these on the excitatory/inhibitory balance and feedforward/feedback balance might complicate the interpretation of our data in relation to various neuropsychiatric effects^1–21^. Despites these limitations, the present data combined with previous findings on microcircuits in the sensory cortex will offer mechanistic insights on the neuromodulatory effects of VNS on the sensory system.

Our main finding of layer-specific VNS effect on AEP amplitude could be explained most parsimoniously by the innervation pattern of nerve terminals; VNS activates the NA, 5-HT and ACh systems^22–24^, all of which innervate superficial cortical layers more densely than deep cortical layers^29–36^. NA^57,64–67^, 5-HT^68–74^ and ACh^75–77^ play crucial roles in gating stimulus-specific plasticity in the sensory cortex. Such gating of plasticity is likely enabled by suppressing the feedback pathway and enhancing the local afferent inputs and feedforward processing^42,78–80^. In addition to such well-established effects of VNS on plasticity^17–19^, our results suggest that VNS plays some different roles in the encoding of ongoing stimulus in the sensory cortex. This neuromodulation contrasts with suppressive effects in superficial layers through top-down attention and active behaviors such as locomotion and whisking^55,58,59^. Canonical motifs of inter-layer microcircuits are also consistent with the notion that the feedforward and feedback inputs are dominantly originated from the superficial and deep layers, respectively^43–47^.

L1 may also mediate VNS modulatory effects in the cortex because nearly all L1 neurons express the ionotropic 5-HT3A receptor and gate a window of thalamocortical disinhibition^81^. Importantly, recent studies imply that L1 plays key roles in gating bottom-up information. While the L1 contains relatively few somata, it features many apical dendrites of local pyramidal neurons and an extensive number of long-range projections that convey contextual, top-down information from higher order thalamic and cortical areas^43,44,46,82–87^. L1 activity can inhibit both excitatory and inhibitory neurons in the L2/3^88,89^. This L1-mediated inhibition tone scales down excitatory and inhibitory inputs in L2/3 but not in L4^58^. A group of GABAergic interneurons in L1 forms unidirectional connections with L2/3 interneurons, which disinhibit L5 dendritic complex spikes, while another group of GABAergic neurons forms mutual inhibitory connections with L2/3 interneurons, which inhibit L5 dendritic complex spikes^46^. L1 interneurons also mediate prolonged inhibition of distal pyramidal dendrites; this inhibition correlates with the strength of the memory trace. Fear conditioning experiment has demonstrated that foot shock induces cholinergic activation in L1, which disinhibits L2/3 according to ongoing contextual information and gates the activity-dependent plasticity in the auditory cortex^90^. Such 5-HT-mediated gating in L1 could also underlie the presently observed VNS-induced modulation.

VNS also modulated SSA in the auditory cortex; however, unlike AEP amplitude, the modulatory effects were not layer-specific. This dichotomy indicates that the click and oddball sequences characterized different modulatory effects, i.e., on neural activation to ascending inputs and on adaptation process, respectively, and suggests that the primitive form of history-dependent prediction is distributed across layers. SSA corresponds to the decrease in the strength of neural responses to a repeated stimulus^48^, the process of which is better characterized just after a deviant stimulus in the oddball paradigm, but not in the click sequence. This decrease usually does not generalize to deviant stimulus. SSA could result in either the depression of the responses to the standard stimuli or in increased responses to deviant stimuli. The latter, for example, could be explained as a violation of expectations set by the repeated stimuli: an indicator of true deviance detection^49,91^. Our experiments showed that VNS decreased SSA via the modulation of the neural responses to standard stimuli. Consistent with this finding, the proportional increase in neural responses was higher for the standard stimuli than for the deviant, resulting in a general decrease in SSA across all layers of the auditory cortex. These results support the idea that VNS may primarily involve the modulation of the cortical ascending input into the auditory cortex instead of modulating a deviance detection mechanism.

In general, GABAergic inhibition modulates SSA in the auditory pathway^92–94^. For example, reducing activities of parvalbumin-positive (PV+) inhibitory interneurons should reduce the contrast between the standard- and deviant-evoked responses^95^. PV+ cells receive strong inhibitory input from SST+ interneurons^96^. On the other hand, the selective upregulation of SST+ interneurons was observed during passive sound exposure that causes a long-lasting reduction, or habituation, in L2/3 pyramidal cells, whereas engagement in auditory tasks diminishes activities of SST+ interneurons and restores the amplitudes of evoked responses to habituated stimuli^97^. Our preliminary results showed that VNS enhanced the gamma-band synchronization in the auditory cortex^98^, suggesting that PV+ interneurons were not deactivated. We thus speculate that VNS inhibits SST+ interneurons through L1^99,100^. In addition, lysergic acid diethylamide (LSD), a 5HT2AR agonist, has been recently shown to reduce neural adaptation to standard stimuli and blunt deviant stimuli-evoked responses^101^. These alterations of neural responses are likely associated with decreases in intrinsic connectivity in A1 and top-down connectivity, which is also the putative effect caused by VNS.

VNS has demonstrated promise as an effective alternative treatment for patients with refractory epilepsy^1,2^ or depression^3,4^. In addition, VNS exerts various neuropsychiatric effects^5–21^; concerning their mechanisms of action underlying these effects, we propose that VNS involves a sensory gain mechanism that primarily affects ascending cortical input. Our results provide insight for future studies on the microcircuits in the cerebral cortex that underpin the effects of VNS. Considering VNS as a sensory gain mechanism could, for example, contribute to the optimization of stimulation parameters during clinical trials. Further studies may improve our understanding of the discrimination of patients (and symptoms) that respond optimally to treatment, the interpretation of the mixed therapeutic results reported in some clinical studies, and ultimately clinical outcomes.

## Methods

### Subjects

Seven 11- to 13-week-old male Wistar rats (body weight: 270 g to 330 g) were used in the experiments. This study was conducted in strict accordance with the “Guiding Principles for the Care and Use of Animals in the Field of Physiological Science” published by the Japanese Physiological Society. The experimental protocol was approved by the Committee on the Ethics of Animal Experiments at the Research Center for Advanced Science and Technology, the University of Tokyo (RAC120103). All surgeries were performed under isoflurane anesthesia, and all efforts were made to minimize animal suffering.

### Surgery and VNS protocol

One week before the main experiments were conducted, a VNS system (VNS Therapy System Model 103 by Cyberonics, Texas) was implanted in the rats under isoflurane anesthesia (3.5–4% at induction and 0.8–2.5% for maintenance). The VNS system consisted of a pulse generator and a spiral electrode; the former was implanted subcutaneously in the back, while the latter was attached to the left vagal nerve. The electrical pulses for VNS were biphasic and charge-balanced to avoid damaging the nerve fibers. The first and second phases had short-time high-amplitude and long-time low-amplitude, respectively, to selectively activate afferent fibers. The current in the first phase was set to 500 µA with the pulse width of 130 μs; and the stimulation frequency, to 10 Hz^102^. VNS of 300 pulses (i.e., 30 s) was applied at 5-min intervals, during which cortical activity induced by VNS was characterized.

### Experimental paradigm

AEPs in A1 were electrophysiologically characterized one week post-implantation. In a click sequence and oddball paradigm, we compared AEPs between pre- and post-VNS condition (Fig. 1). To avoid any residual effect of VNS on the electrophysiological recordings, the pre-VNS condition always preceded the post-VNS condition; VNS was applied once the blocks of the pre-VNS condition trials had been completed. Three sessions of VNS were conducted before beginning the first session of the post-VNS condition. Each block of auditory stimulation (e.g., the click sequence and the oddball paradigm) was shorter than 5 min to be completed within the 5-min interval of VNS, and was preceded by 30 s of VNS under the post-VNS condition. Pre- and post-VNS conditions featured different orders of experimental paradigms to assign the highest priority to the characterization of VNS effects on the most distinct AEPs in the click sequence.

*Click sequence*: A click was defined as a monophasic positive sound wave with a duration of 20 ms. Clicks were played in a block of trials at a rate of 1 Hz. A block of trials consisted of 240 clicks, and the total duration of a block was 4 min (Fig. 1).

*Oddball paradigm:* The oddball paradigm employed a sequence of standard and deviant stimuli^48–53^. Characterized by different tone frequencies, the standard and deviant stimuli were presented at discrepant rates: 90% for the former and 10% for the latter. We conducted two sessions, each with two blocks of trials. In the first session, we used 10- and 16-kHz tones; in the second session, 20- and 32-kHz tones. In the second block of each session, the tones frequencies of standard and deviant stimuli were inverted from those in the first block to compare the standard- and deviant-evoked AEPs with an identical frequency. For example, in the first block of the first session, 10-kHz tones served as standard stimuli and 16-kHz tones as deviant, while the second block employed 16-kHz tones as standard stimuli and 10-kHz tones as deviant. The test stimuli were tone bursts with a 5-ms rise/fall, 90-ms plateau, and 60 dB sound pressure level (in decibels with respect to 20 μPa; SPL). In each block, 450 standard stimuli and 50 deviant stimuli were randomly presented every 500 ms, taking 250 s in total.

### Electrophysiological recordings

On the day of the experiment, the second surgery was performed to conduct the electrophysiological recordings in a sound-proof room. The experimental procedures employed in the present study have been previously reported^52–54^. Rats were anesthetized with isoflurane in conjunction with air (3% for induction and 1–2% for maintenance) and were held in place with a custom-made head-holding device. A small craniotomy was performed near the bregma landmark to embed a 0.5-mm-thick integrated circuit socket as a reference electrode with electrical contact to the dura mater. The right temporal muscle, cranium, and dura overlying the A1 were surgically removed, and the exposed cortical surface was perfused with saline to prevent desiccation. The right eardrum (ipsilateral to the exposed cortex) was ruptured and waxed to ensure unilateral sound input from the ear contralateral to the exposed cortex. The speaker used for acoustic stimulation (Technics EAS-10TH800, Matsushita Electric Industrial Co. Ltd., Kadoma-shi, Osaka, Japan) was positioned 10 cm from the left ear, contralateral to the exposed cortex.

We first confirmed the location of the A1 through surface microelectrode recording^52,53^. Laminar AEPs were then recorded from the A1 using Cerebus Data Acquisition System (Cyberkinetics Inc., Salt Lake City, UT, USA) and a custom-made microelectrode array (NeuroNexus Technologies, Ann Arbor, MI, USA). The microelectrode array had three shanks; each was 6 mm long and 50 μm thick, and the inter-shank distance was 500 μm. Each shank had 32 recording sites: 15 distal sites in the thalamus – i.e., the medial geniculate body (MGB) – and 17 proximal sites in the cortex – i.e., the A1 (Fig. 2a). The distance between the most proximal site in the MGB and the most distal site in the A1 was 1200 μm. The diameter of the recording sites was 30 μm, and the inter-electrode spacing was 120 μm. An array of three shanks was inserted perpendicularly to the cortical surface of the A1 (approximately 5.0–6.0 mm posterior to the bregma). A needle electrode was subcutaneously inserted into the right forepaw and used as a ground. Multi-unit activities (MUAs) and LFPs were measured with respect to the reference electrode near the bregma. MUAs were recorded at a sampling rate of 30 kHz with a filter between 250 and 7500 Hz, and MUA spikes were detected online by threshold-crossing (set to 5.13–5.35 times the RMS of the signal). LFPs were obtained at a sampling rate of 1 kHz with a filter between 0.3 and 500 Hz.

### Data analysis

#### Identification of layers in auditory cortex

Based on the AEPs recorded in response to tone bursts with a frequency of either 8, 16, or 32 kHz at 60 dB SPL (5 ms rise/fall, 90 ms plateau), we identified the location of the electrodes across the layers in the A1 (Fig. 2b). Each tone was presented 240 times in a block at a rate of 1 Hz. The grand average of AEPs with the largest responses were used to compute a conventional pattern of current source density (CSD): the spatial second derivative estimate of the laminar AEP time series^51,54,103^. CSD was calculated with the following formula: ${V}_{u}+{V}_{l}\mbox{--}2{V}_{o}/{\varDelta x}^{2}$, where *V*_*o*_ indicates the AEPs at a given depth, *V*_*u*_ and *V*_*l*_ are the AEPs at the upper and lower adjacent sites, respectively, and *Δx* indicates the distance between the recording sites (i.e., 120 μm). Based on the CSD, the recording sites were classified into separate layers of the A1 according to our empirical criteria determined by the previous works^51,54,103^: layers 1, 2/3, 4, 5 or 6 (L1, L2/3, L4, L5, or L6). L1 was defined as the uppermost site of the source. For L2/3, a single site with a sink followed by a short source was chosen. L4 was defined as a site with the earliest sink and the above adjacent site (two sites). L5 was defined as two successive sites with sources below L4. Weak sinks could be found in the deeper sites, of which the second deeper site was labeled as L6.

#### Identification of the characteristic frequency

The frequency responsive area (FRA) was characterized from MUA at each recording site to confirm that the tested recording site was located either in the A1 or MGB^54^. The test stimuli were tone bursts (5 ms rise/fall and 20 ms plateau) with frequencies ranging from 1.6–64 kHz with 1/3 octave increments and intensities varying from 20–80 dB SPL in 10-dB increments. A total of 126 tone bursts were used to characterize FRA, where tone-evoked discharge rates were quantified with respect to the test frequency and intensity. Each test tone was repeated 20 times in a pseudorandom order with an inter-tone interval of 600 ms. Based on the FRA, the characteristic frequency (CF) was determined as the frequency at which test tones evoked a response for the lowest intensity or the largest response at 20 dB SPL. The shank was typically located in mid-to-high CF regions of the A1. We computed the median of the CF across contacts and classified the shanks into two groups according to CF: mid CF, 13–20 kHz, and high CF, 25–40 kHz. In oddball paradigms, AEPs recorded in response to 16 kHz and 32 kHz were characterized in the mid and high CF shanks, respectively.

#### Characterization of AEPs

To investigate the effects of VNS on the AEPs across cortical layers, we compared the grand average of the AEPs across trials at a given layer between pre-VNS and post-VNS conditions. Because two sites were used to represent L4 and L5 (Fig. 2b), AEPs were averaged across the representative sites in these layers. We also quantified the effect of VNS on SSA for each recording site as the SSA index: ${SI}=({Pd}-{Ps})/({Pd}+{Ps})$, where Ps and Pd are the peak amplitudes of the standard and deviant AEPs, respectively. SI reflects the proportional changes in the neural responses to standard and deviant stimuli. SI was characterized by one of test frequencies that most match the CF at a given recording site.

*Statistical tests:* For AEP amplitude and SI, after confirming normality (Lilliefors test, p > 0.05), two-way repeated measures ANOVA was performed using VNS conditions (pre vs post) and layers (L1, L2/3, L4, L5 and L6) as factors. The effect sizes of VNS on the AEP amplitudes and SI were also quantified by calculating Cohen’s d across cortical layers.
